# Whole mitochondrial genome sequencing highlights mitochondrial impact in gastric cancer

**DOI:** 10.1038/s41598-019-51951-x

**Published:** 2019-10-31

**Authors:** Giovanna Chaves Cavalcante, Anderson N. R. Marinho, Ana Karyssa Anaissi, Tatiana Vinasco-Sandoval, André Ribeiro-dos-Santos, Amanda Ferreira Vidal, Gilderlanio S. de Araújo, Samia Demachki, Ândrea Ribeiro-dos-Santos

**Affiliations:** 1 Laboratório de Genética Humana e Médica, Universidade Federal do Pará, Belém, 66075-970 Brazil; 2 Núcleo de Pesquisas em Oncologia, Universidade Federal do Pará, Belém, 66073-005 Brazil

**Keywords:** Medical genomics, Cancer genetics

## Abstract

Mitochondria are organelles that perform major roles in cellular operation. Thus, alterations in mitochondrial genome (mtGenome) may lead to mitochondrial dysfunction and cellular deregulation, influencing carcinogenesis. Gastric cancer (GC) is one of the most incident and mortal types of cancer in Brazil, particularly in the Amazon region. Here, we sequenced and compared the whole mtGenome extracted from FFPE tissue samples of GC patients (tumor and internal control – IC) and cancer-free individuals (external control – EC) from this region. We found 3-fold more variants and up to 9-fold more heteroplasmic regions in tumor when compared to paired IC samples. Moreover, tumor presented more heteroplasmic variants when compared to EC, while IC and EC showed no significant difference when compared to each other. Tumor also presented substantially more variants in the following regions: MT-*RNR1*, MT-*ND5*, MT-*ND4*, MT-*ND2*, MT-*DLOOP1* and MT-*CO1*. In addition, our haplogroup results indicate an association of Native American ancestry (particularly haplogroup C) to gastric cancer development. To the best of our knowledge, this is the first study to sequence the whole mtGenome from FFPE samples and to apply mtGenome analysis in association to GC in Brazil.

## Introduction

Mitochondria are cytoplasmic organelles that perform major roles in cell operation, including energy generation through oxidative phosphorylation (OXPHOS), cell death, calcium levels control, lipid homeostasis and metabolic cell signaling^1^. These organelles have their own genome (mtGenome), with 16,569 bp of length and 37 genes, of which 13 are protein-coding genes involved in OXPHOS, 22 are transfer RNA (tRNA) genes and two are ribosomal RNA (rRNA) genes^2,3^. It also presents a non-coding control region known as displacement loop (D-loop), essential for replication and transcription regulation^4^. There are many copies of mitochondria in each cell and such copies may present different alleles for the same variant, a state called heteroplasmy.

It is well-known that mitochondrial DNA (mtDNA) is more susceptible to alterations in comparison to nuclear DNA^5–7^. These alterations may lead to mitochondrial dysfunction, which in turn may account for cellular deregulation due to DNA repair defects, leading to the development of different diseases, such as cancer^8^. It is notable that hallmarks of cancer (*i*.*e*. abilities acquired by tumor during carcinogenesis to survive and proliferate) include energy deregulation and evasion of cell death, both directly related to mitochondrial function^9^. In fact, many studies have shown an association of mtDNA instability and heteroplasmy to different types of cancer^4,10–15^.

One of these types of cancer is gastric cancer (GC), which is currently the fifth most incident and the third most lethal type of cancer worldwide^16^. In Brazil, GC is also one of the most frequent and aggressive types of cancer, being the sixth most incident and the fifth most lethal cancer^16^. This is even more alarming in the North region of Brazil, where GC is the second most incident type of cancer among men and the fifth among women^17^, probably due to eating habits and genetic background of the population.

Regarding genetic ancestry, it is important to highlight that, in addition to nuclear DNA, mtDNA also provides such information through haplogroups, which are basically groups of haplotypes. These provide information about women migration and have also been associated to development and outcome of different diseases, including GC^18–21^.

Moreover, GC usually presents an unfavorable clinic evolution because of nonspecific symptoms at early stages, leading to late diagnosis and a poor prognosis^22^. Thus, it is crucial to search for genetic markers that would allow an earlier detection and improve patient outcome.

In the last decade, with the advent of new technologies such as Next-Generation Sequencing (NGS), one focus of oncologic research has been high-throughput analyses of human genome related to cancer development. However, to this date, not many studies have investigated the association of mtGenome alterations to cancer, especially GC. For instance, a recent review has pointed out 16 studies associating mtDNA alterations to gastric cancer, but none involved NGS approaches^23^.

In this study, we sequenced the whole mtGenome in order to assess and compare variants and their heteroplasmy levels in FFPE samples from gastric cancer patients (paired samples, *i*.*e*. samples of both tumor and non-tumoral tissues) and cancer-free individuals from the North region of Brazil. To the best of our knowledge, this is the first study to perform such analysis of gastric cancer in Brazil.

## Results

After processing the mtGenome sequences with the adequate mapping scores (Q30), the average depths of coverage were 195x and 36x for tumor samples and internal control (IC) samples, respectively. For external controls (EC), the average depth of coverage was 351x. All samples with low quality were excluded. Table 1 presents the sample size of all groups for each analysis.

**Table 1:** Sample number of each group in the different comparison analyses.

	CASE		CONTROL
	Tumor	Internal Control (IC)	External Control (EC)
Initial sample number	20	20	50
After processing	13	11	47
***Paired analyses of variant distribution***			
Paired analysis	8	8	—
***Heteroplasmy analyses***			
Initial sample number	13	9	40
Paired analyses	6	6	—
General analyses	13	9	35
***Mitochondrial haplogroup analyses***			
Sample number	—	11	47

Out of the 20 sequenced sample pairs of GC patients, only eight had both samples with enough coverage for a paired comparison. In another eight patients, only one of their samples (five tumors and three internal controls) had enough coverage to be included in further analyses. The remaining four pairs did not pass our quality standards and were excluded from all analyses. As for external controls, 47 samples had enough coverage to be included in the general analyses.

It is noteworthy that (i) mean age was of 66 years old in case group and 41 years old in EC group (Mann-Whitney test, p < 0.001); and that (ii) case group was composed of 77% male and 23% female, while control group was composed of 28% male and 72% female (Chi-squared test, p = 0.002).

Therefore, we evaluated if sex or age range (≥70, 60–69, <60 years-old) in the tumor samples were associated to the number of variants (Kruskal-Wallis Test; H 1.52, 3.18; P 0.467 and 0.075, respectively), primary tumor location (χ^2^ 13.07, 10.18; P 0.364 and 0.117), tumor degree of differentiation (χ^2^ 6.17, 0.82; P 0.187 and P 0.662), pathogenic staging pT (χ^2^ 9.19, 5.68; P 0.514 and 0.339) and pN (χ^2^ 8.44, 6.43; P 0.392 and P 0.169) and found no association. Similarly, we did not find any dependency of age range (≥60, 50–59, 40–49, 30–39, 20–29, <20 years-old) or sex to the number of variants of EC (Kruskal-Wallis Test; H 5.22, 0.03; P 0.390 and 0.856, respectively). Thus, age and sex were disregarded in further analyses.

### Paired sample analyses

When we compared the mtGenome of each of the eight paired samples from gastric cancer patients, we found that, in most cases, tumor samples presented more variants than their respective internal control samples (up to 5.5-fold more variants). Table 2 shows the number of variants by pair and the genes in which most variants were found. The full list of variants and proportions is found in Supplementary Material (Table S1).

**Table 2:** Number of variants (N) and the genes in which most variants were found in each pair of tumor and internal control samples from the same individuals.

Pair	Tumor-exclusive variants		Internal control exclusive variants		Shared variants	
	N	MT-Regions	N	MT-Regions	N	MT-Regions
1	22	DLOOP1 (22.7%) DLOOP2 (22.7%) ND5 (18.2%)	4	ND6 (50%)	4	ATP6 (25%) ND4 (25%) ND5 (25%) RNR1 (25%)
2	13	DLOOP2 (38.5%) ND5 (15.4%)	13	ATP6 (23.1%)	3	CO3 (33.3%) CYB (33.3%) DLOOP1 (33.3%)
3	16	DLOOP1 (31.3%) ND5 (31.3%)	10	DLOOP1 (40%)	6	ATP6 (16.7%) CO1 (16.7%) CO3 (16.7%) DLOOP1 (16.7%) ND4 (16.7%) ND5 (16.7%)
4	12	ND4 (25%) DLOOP2 (16.7%)	7	ND5 (42.9%)	4	ATP6 (25%) CYB (25%) ND4 (25%) ND5 (25%)
5	24	DLOOP2 (16.7%) DLOOP1 (12.5%)	5	ND5 (60%)	4	DLOOP1 (50%)
6	4	ND5 (50%)	15	ATP6 (20%)	13	CO3 (23.1%) CYB (23.1%) DLOOP1 (23.1%)
7	14	DLOOP1 (28.6%) ND1 (21.4%)	6	ATP8 (16.7%) CO1 (16.7%) DLOOP2 (16.7%) ND2 (16.7%) RNR1 (16.7%) RNR2 (16.7%)	3	CYB (33.3%) ND5 (33.3%) RNR1 (33.3%)
8	11	ND4 (18.2%) DLOOP2 (18.2%) RNR1 (18.2%)	8	ND5 (37.5%) DLOOP1 (25%)	3	DLOOP1 (66.7%) CYB (33.3%)

Considering exclusively single variants (*i*.*e*. each variant individually), there were 50 variants present only in tumor samples, while eight were present only in internal controls. These 50 variants were distributed in 17 regions, but mostly in four regions: MT-*DLOOP1* (16%), MT-*ND5* (16%), MT-*ND4* (10%) and MT-*DLOOP2* (10%). As for variants only found in internal controls, most of them were located in MT-*ND5* (25%).

**Figure 1:**
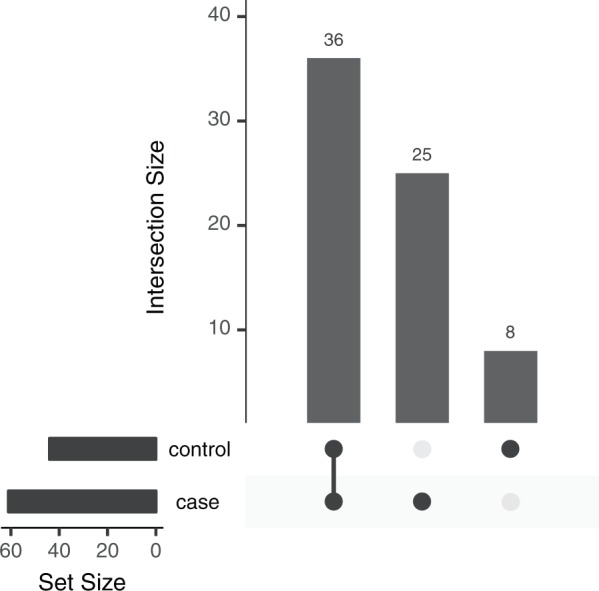
Number of exclusive and shared variants found in the paired samples (tumor and internal control) of gastric cancer patients. Groups are represented by the darker dots. Set size is the sum of variants for each group.

By grouping all paired samples from tumor and IC, we found 25 variants that were exclusive to tumor samples and eight variants that were exclusive to internal control samples, as well as 36 variants that were shared by both groups (Fig. 1). The distribution by region and position of these variants is shown in Supplementary Material (Table S2).

Hence, tumor samples presented more than 3-fold the number of exclusive variants in comparison to their paired internal control samples. This notable number of variants that were exclusive to tumor was distributed in 13 genes, of which three (MT-*ATP8*, MT-*ND4L* and MT-*TG*) were only affected in this group. The variants exclusive to internal control samples were distributed in seven genes and the shared variants were distributed in 14 genes, of which MT-*CO3* and MT-*RNR1* were affected in both groups.

In addition, the genes with more variants were: (i) for tumor-exclusive variants, MT-*DLOOP1* and MT-*DLOOP2* (with 16% each); (ii) for variants exclusive to internal control, MT-*ND5* (with 25%); and (iii) for variants shared by both groups, MT-*DLOOP1* (with 22%) and MT-*ND5* (with 19%).

**Figure 2:**
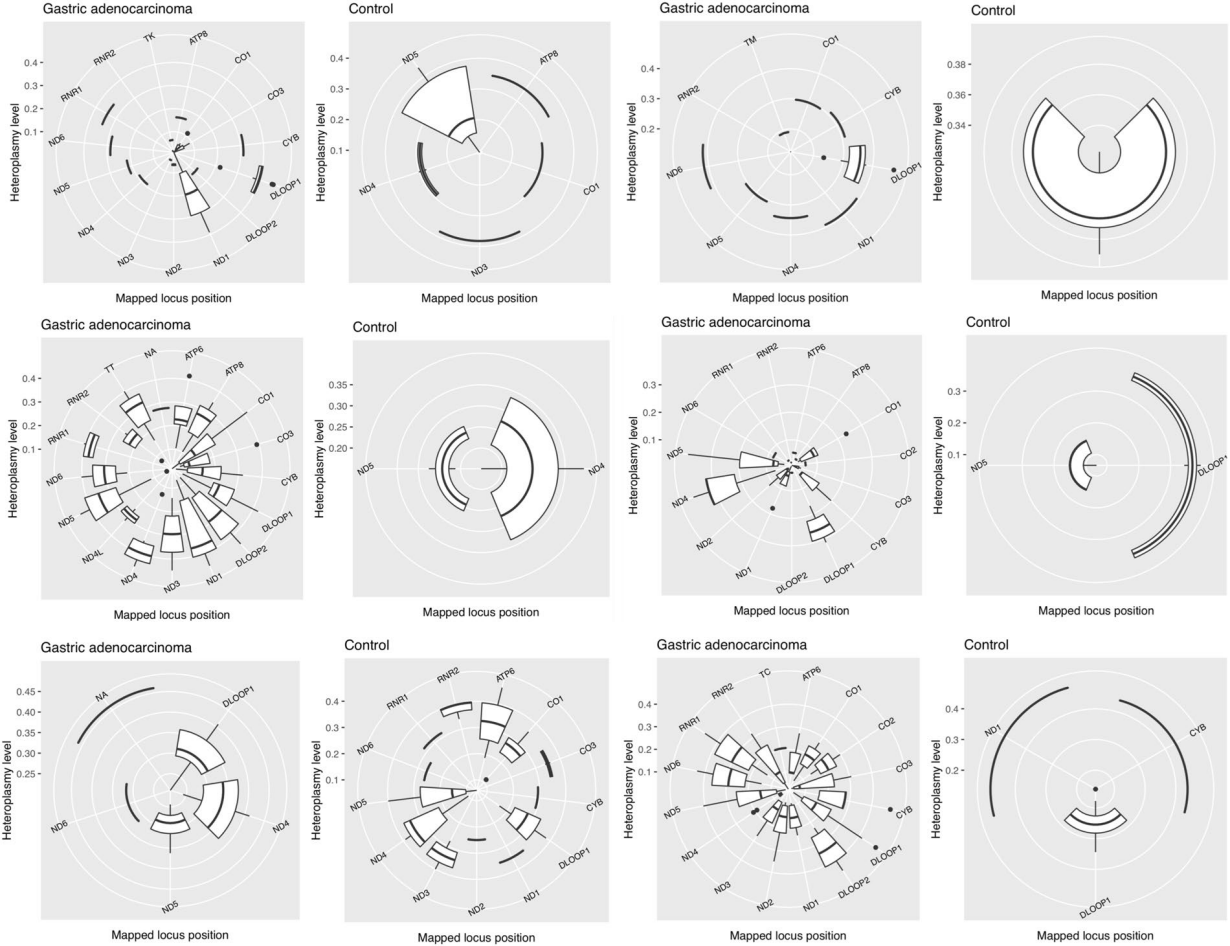
Heteroplasmy levels for each pair of samples from gastric cancer patients, both tumor (gastric adenocarcinoma) and internal control (control). Each boxplot represents the distribution of heteroplasmy in different mitochondrial genes. Non-applicable (NA) represents unidentified regions.

When comparing heteroplasmy, six out of the eight pairs matched our standards and were included in this analysis. We found that, with one exception, tumor presented notably more heteroplasmic regions than their paired internal control (3 to 9-fold times more; Fig. 2).

Among the heteroplasmic regions, the most commonly affected were MT-*DLOOP1*, MT-*ND4* and MT-*ND5*. It is noteworthy that these three, along with MT-*ND6*, were heteroplasmic in all tumor samples, but not in all internal controls. Four other genes were heteroplasmic in almost all tumor samples, but they were not as frequently affected among internal control samples (MT-*CO1*, MT-*CYB*, MT-*ND1* and MT-*RNR2*). Additionally, seven other regions were heteroplasmic only in the tumor (MT-*CO2*, MT-*DLOOP2*, MT-*ND4L*, MT-*TC*, MT-*TK*, MT-*TM* and MT-*TT*), and there were no genes that were heteroplasmic in the internal control group but not in the tumor group.

### General heteroplasmy analysis

When comparing heteroplasmy levels between all groups of samples (tumor, internal control and external control), we included all tumor (N = 13), internal control (N = 9) and external control (N = 40) samples that met our quality control standards (see Table 1). In addition to the quality scores, only variants with at least 5% heteroplasmy levels were considered for this analysis, in order to control possible artifacts and false-positives due to the limited coverage of the samples^24,25^.

Using this criterion, we assessed heteroplasmy levels of all three groups. Aiming a fair comparison of heteroplasmy levels, we evaluated the presence of the same distinct 630 variants in all samples considering a heteroplasmy level of 1% when absent or under 5%.

The log heteroplasmy levels were compared using a mixed linear regression assuming a fixed effect of the group and a random effect of the individuals. This model was better fitted to the data than a model without the group effect (Wald test χ^2^ 10.11; P 0.006). The pairwise comparison (with the p-value adjusted according to Tukey) showed that the tumor group presented a significantly higher level when compared to internal group (*t* 2.60; P 0.025) and external group (*t* 3.04; P 0.006) both with statistical power above 73%. These control groups did not present statistically significant difference between each other (*t* −0.42; P 0.906).

Considering the previous analysis, the amount of heteroplasmic variants may be more important to tumor development than heteroplasmy levels of variants *per se*. In fact, out of 1,664 heteroplasmic variants found after filtering, 653 variants were found in tumor subgroup (representing 39% of the total), 152 in internal control subgroup (9%) and 859 in external control subgroup (52%). These proportions are very expressive, considering that tumor samples account for about only 1/3 of the number of external controls and they still showed almost half of all heteroplasmic variants.

**Figure 3:**
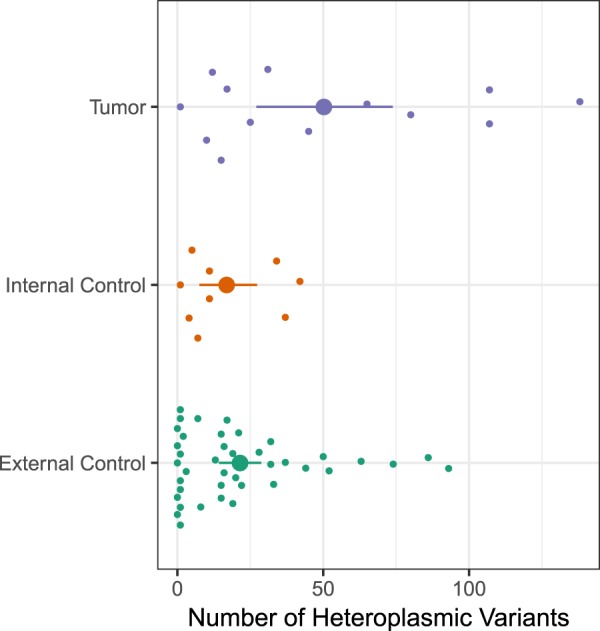
Distribution of heteroplasmic variants in the studied groups: tumor, internal control and external control. Each small dot represents the mean of heteroplasmic variants of a sample. The average number of heteroplasmic variants is also represented for each group.

Further, individual’s number of heteroplasmic variants average (*i*.*e*. total of heteroplasmic variants divided by number of samples) was of 50.23 for tumor, 16.89 for internal control and 24.54 for external control. Thus, tumor samples presented nearly 3-fold more heteroplasmic variants than internal control samples (Fig. 3). Then, a Poisson regression considering group effects was applied and it showed a significant difference between these groups (ANOVA F 5.46; P 0.007), with a statistical power above 90%.

In Fig. 3, it is possible to see that, even though the external control group was composed of more samples, most of them showed less heteroplasmic variants in comparison to tumor samples. Proportionally, internal control group also showed many samples with less heteroplasmic variants than tumor group. In addition, about half of tumor group presented samples with a high number of heteroplasmic variants, including some samples with more than 2-fold the mean tumor’s heteroplasmy variants found (approximately 50 variants).

This difference became even more evident in the pairwise comparison of number of heteroplasmic variants between all groups using a post-hoc Tukey test. Tumor group presented a statistically significant difference when compared to internal (P 0.027) and external (P 0.008) controls with a statistical power above 70%. However, when both controls were compared no significant difference was found (P 0.903).

Regarding location of the observed heteroplasmic variants, heteroplasmy levels were observed in all protein-coding genes and in almost all non-protein-coding genes – appearing in all DLOOP and rRNA genes, but not in all tRNA genes. It is noteworthy that two heteroplasmic variants were observed in unidentified regions in external control and tumor samples and that they are probably in tRNA genes due to location. These unidentified regions were excluded from further analyses.

**Figure 4:**
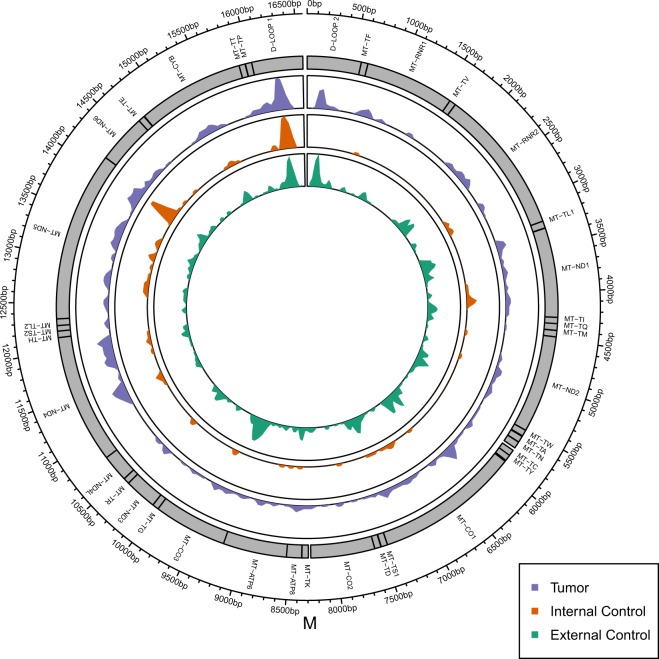
Distribution of heteroplasmic variants in the mitochondrial genome for all groups: tumor, internal control and external control. Peaks represent the number of variants. Genomic length and gene division are also shown.

Figure 4 shows a representation of all protein-coding and non-coding regions throughout 16,569 bp of the mtGenome. This graph supports our previous result that tumor group presents most heteroplasmic variants, followed by external control and internal control groups.

**Figure 5:**
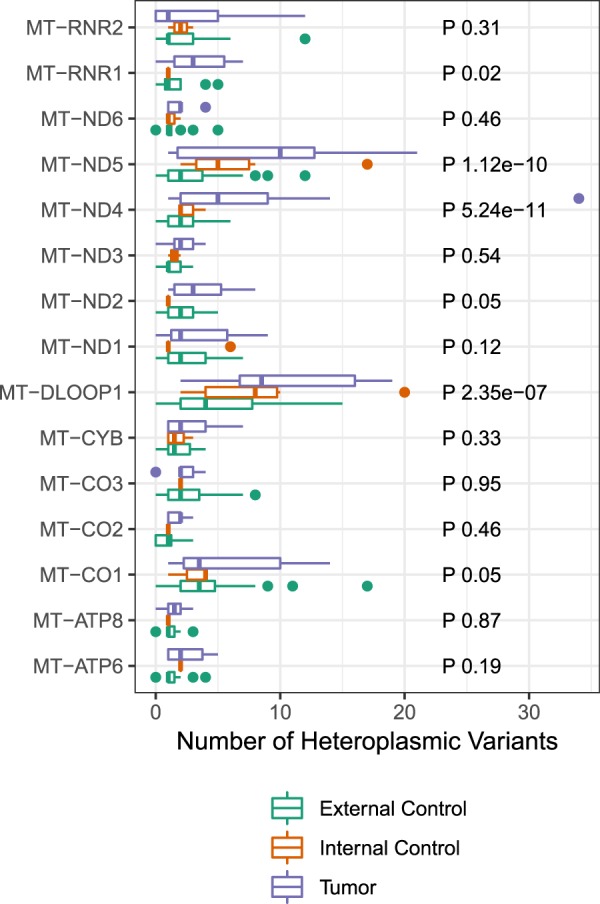
Joined comparison of all three groups regarding mitochondrial genes with heteroplasmic variants, with the respective p-value obtained with Poisson regression.

Moreover, out of the 37 genes in mtGenome, 32 were heteroplasmic in external control group, 28 in tumor group and 15 in internal control group. Hence, in order to compare heteroplasmic regions between all three groups, we analyzed 15 regions affected in all of them. Among such regions, 12 were protein-coding and three were non-protein-coding genes (one DLOOP and two rRNA regions) (Fig. 5). In this analysis, we observed a statistically significant difference between all groups, with tumor group presenting more heteroplasmic variants in MT-*RNR1*, MT-*ND5*, MT-*ND4*, MT-*ND2*, MT-*DLOOP1* and MT-*CO1*.

### Mitochondrial haplogroup analyses

Furthermore, we assessed haplogroup information of all samples in order to investigate a possible association with GC. In some cases, tumor samples presented a different haplogroup than their paired samples, indicating that the tumors might not be an accurate ancestry informative tool, especially considering that MT-DLOOP presented a particularly high rate of variants in this group and these regions are the most informative of maternal ancestry^26^. Thus, all tumor samples were disregarded from haplogroup analyses and the case group was be represented only by the internal control.

Considering both case (internal control) and control (external control) groups, we observed 17 macro-haplogroups, out of which there were four Native American (A, B, C and D), four European (H, HV, J and V), six African (L0, L1, L2, L3 and L3e) and four Asian (M, N, R and Y) haplogroups (Table 3). Native American haplogroups were the most frequent in both case (36.4%) and control (44.6%). Following, control group presented about the same frequency rate for both European and African haplogroups (25.6% each), while case group presented a similar rate for European (27.3%), but not for African haplogroups (18.2%). Asian haplogroups were around 4-fold more frequent in cases (18.2%) than in controls (4.2%).

**Table 3:** Distribution of macro-haplogroups in case and control groups.

Mitochondrial Ancestry	Macro-haplogroups	General (%)	Case (%)	Control (%)
Native American	A	7.0	0.0	8.5
	B	8.6	0.0	10.6
	C	20.7	36.4	17.0
	D	7.0	0.0	8.5
European	H	13.8	18.2	12.8
	HV	3.4	9.1	2.1
	J	3.4	0.0	4.3
	V	5.2	0.0	6.4
African	L0	1.7	0.0	2.1
	L1	13.8	9.1	15.0
	L2	1.7	0.0	2.1
	L3	1.7	9.1	0.0
	L3e	5.2	0.0	6.4
Asian	M	1.7	9.1	0.0
	N	1.7	0.0	2.1
	R	1.7	0.0	2.1
	Y	1.7	9.1	0.0

The most common haplogroup was C (Native American ancestry) in both groups, but it is interesting that case has more than 2-fold the frequency of the control (36.4% and 17.0%, respectively). In case group, the second most common haplogroup was H (European ancestry), with 18.2%, followed by the other five haplogroups with 9.1% of presence each. In control group, haplogroup L1 (African ancestry) is the second most common, with 15%, followed by haplogroup H, with 12.8%, and the other haplogroups.

**Figure 6:**
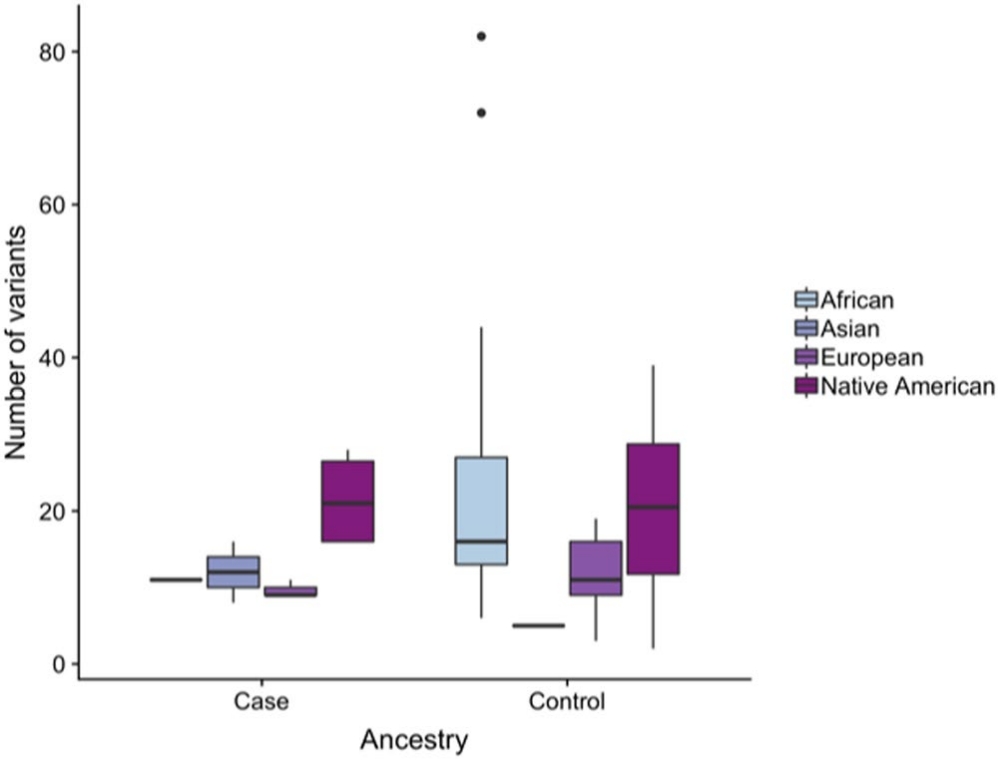
Number of variants in the four ancestries (African, Asian, European and Native American) of both case and control groups.

In addition, our results showed a difference in the distribution of variants by mitochondrial ancestry (Fig. 6). Firstly, we compared it within cases using ANOVA and found that 53% of all variants were in individuals from Native American haplogroups, followed by European (18%), Asian (15%) and African (14%) haplogroups (F 4.48; P 0.047). As for control group, individuals from Native American haplogroups also presented the majority of variants (46%), followed by African (36%), European (16%) and Asian (2%) haplogroups (F 2.57; P 0.066). When we compared case and control there was no significant difference (F 0.93; P 0.338).

## Discussion

In this study, we investigated the association of mitochondrial variants with gastric cancer in individuals from a population of the Brazilian Amazon. This was done through whole mtGenome sequencing of FFPE samples from patients and cancer-free individuals. To the best of our knowledge, this is the first study to successfully sequence mtGenome from FFPE samples.

This is particularly important because DNA sequencing from this type of material can be challenging. Although good for long-term storage of tissue samples, the FFPE process may affect the material integrity so that the extracted DNA is generally of low quality, often leading to the loss of samples. However, some studies performing NGS have shown that DNA extracted from FFPE tissue samples can still provide results in concordance to frozen tissues^27–29^. The challenge presented by FFPE samples is probably the reason for the loss of samples and for the limited coverage in our study. In spite of that, we were able to successfully sequence the mtGenome, obtaining interesting results.

When we compared the samples from the same eight gastric cancer patients, *i*.*e*. paired tumor and internal control samples, we observed significantly more somatic variants present in tumor samples than in the respective internal control samples (up to 5.5-fold more), a kind of pattern that has been reported in other types of cancer, such as colorectal cancer and penile cancer^11,30^. In addition, studies comparing mtDNA variants and genomic instability of different types of cancer reported that gastric cancer was among the types with more somatic variants and genomic instability with a tumor-specific pattern^10,25^.

Regarding the mitochondrial genes in which most of the tumor-exclusive variants were found, we observed that MT-*DLOOP2*, MT-*DLOOP1* and MT-*ND5* were among the regions with more variants in at least half of the tumor samples included in this analysis (Table 2). There was no similar pattern observed when we considered the regions with more variants exclusive to internal control. As for shared variants, they were similarly distributed among different genes. Additionally, there was a mean of 2.43-fold increase in the number of genes with exclusive variants when we compared tumor and internal control samples. It is noteworthy that most of these variants are present in more than one sample and are counted as such in this analysis.

Therefore, when we considered single variants only, there were 50 variants present only in tumor samples and most of them was distributed in four regions – MT-*DLOOP1* (16%), MT-*ND5* (16%), MT-*ND4* (10%) and MT-*DLOOP2* (10%) –, while there were eight variants present only in internal control samples, of which most was found in MT-*ND5* (25%). This suggests that, in tumor, there is an increase not only in the number of variants, but also in the regions in which such variants occur.

This greater presence of variants in tumor samples was reinforced when we grouped and compared the eight pairs. In this analysis, we found 25 tumor-exclusive somatic variants and eight somatic variants that were exclusive to internal control samples, as well as 36 shared variants. While some variants that were considered somatic in the pair-by-pair comparison were then considered germline, tumor samples still showed 3-fold more variants than internal control samples.

Such tumor-exclusive variants were distributed in 13 genes, of which three (MT-*ATP8*, MT-*ND4L* and MT-*TG*) were only observed in this group. Only a few studies have reported variants in MT-*ATP8*^31,32^ and MT-*ND4L*^31,33–35^ in different types of cancer and none was in gastric cancer. No studies were found associating variants in MT-*TG* to cancer.

Furthermore, two genes (MT-*CO3* and MT-*RNR1*) only presented shared variants, which are probably not involved in tumorigenesis. However, five genes (MT-*CO1*, MT-*CYB*, MT-*DLOOP1*, MT-*ND1* and MT-*ND5*) not only presented germline variants, but also different somatic variants exclusive to tumor and internal control groups, indicating a possible association of such genes and variants with tumor development. It is noteworthy that, among these genes, MT-*DLOOP1* and MT-*ND5* stand out for presenting more germline variants than the others.

In addition to the shared variants, MT-*ND5* also presents a high rate of exclusive variants not only in tumor samples but also in internal control samples, suggesting that this gene could also be altered in cells in other adjacent organs of cancer patients, contributing to possible tumor advances. Regardless, MT-*ND5* encodes a core subunit of Complex I, essential for electron transport from NADH to ubiquinone in the mitochondrial respiratory chain, so that variants in this gene and others related to this process may lead to impairment in OXPHOS and an increase of reactive oxygen species (ROS) generation, which can contribute to cancer proliferation and metastasis^36,37^. In fact, a high rate of mutations in MT-ND5 has been reported in esophageal cancer^38^.

Moreover, the regions with more tumor-exclusive variants were the MT-*DLOOP1* and MT-*DLOOP2* (16% each). This corroborates previous studies that associated an increase of variants in such control regions to the development of different types of cancer, including hepatocellular carcinoma^39^, brain tumor^40^, oral squamous cell carcinoma^41^, colon cancer^42^ and gastric cancer^43^. Further, a study have associated five variants in these genes with gastric cancer, including A73G and T16519C^44^, which we found frequently in our tumor samples but not in internal control samples.

We also obtained interesting results in the paired analysis of heteroplasmy levels. In most cases, tumor presented notably more heteroplasmic regions than the paired internal control (3 to 9-fold). About these heteroplasmic regions, we highlight that: (i) the four most commonly heteroplasmic genes in tumor samples were not heteroplasmic in all internal control samples; (ii) four genes were heteroplasmic in almost all tumor samples, but not as frequent in internal control genes; (iii) seven genes were heteroplasmic only in tumor samples; and (iv) there were no genes heteroplasmic only in the internal control group. These results are suggestive of an involvement of such heteroplasmic variants in GC development.

This was reinforced in our analyses comparing heteroplasmy distribution between all groups of samples (tumor, internal control and external control). When we compared heteroplasmy level means, the number of variants was different between groups, so we normalized the number of single heteroplasmic variants in all groups and found a statistically significant difference from tumor to internal control (P 0.025) and external control (P 0.006). These control groups did not present such difference between each other (P 0.906).

Therefore, the number of heteroplasmic variants might be as important or even more important to cancer development than heteroplasmy levels *per se*. In the individual heteroplasmy mean comparison, we observed that individual tumor samples presented almost 3-fold more heteroplasmic variants than internal control samples. A study conducted with patients of hepatocellular carcinoma also reported that tumor samples presented more heteroplasmic variants and a higher heteroplasmy ratio than their matched cancer-free samples^45^.

Moreover, tumor group presented 39% of all heteroplasmic variants, while internal control group presented 9% and external control group presented 52%. This is especially interesting given that tumor group had about a third of the number of external control samples, but about half of tumor group was composed of samples with a high number of heteroplasmic variants, as seen in Fig. 3. When compared to the control groups, tumor group presented a statistically significant difference regarding this distribution of heteroplasmic variants (P 0.027 for internal control and P 0.008 for external control). There was no significant difference between both controls (P 0.903).

As for location of heteroplasmic variants, out of the 37 mitochondrial genes, 32 presented variants in at least one group, out of which 15 regions carried such variants in all three groups, allowing the comparison shown in Fig. 5. Among these regions, 12 were protein-coding and three were non-protein-coding genes (DLOOP and rRNA only). Further, the higher rate of heteroplasmic variants in tumor groups was statistically significant in six of such regions: MT-*RNR1*, MT-*ND5*, MT-*ND4*, MT-*ND2*, MT-*DLOOP1* and MT-*CO1*.

In the current specialized literature, not many studies were found associating heteroplasmic variants to the development of gastric cancer, regardless of mitochondrial genomic location, including the six genes above. A study performed showed an association of heteroplasmic variants in DLOOP regions to gastric cancer development in a Chinese population^46^. Another study have indicated homoplasmic and heteroplasmic variants in mitochondrial 12 S rRNA (encoded by MT-*RNR1*) to the development of intestinal-type gastric cancer^47^. No studies were found associating heteroplasmic variants in the four other genes to GC development.

In the mitochondrial haplogroup comparison between case and control, we observed 17 macro-haplogroups, distributed in four main ancestries: Native American (A, B, C and D), European (H, HV, J and V), African (L0, L1, L2, L3 and L3e) and Asian (M, N, R and Y).

Our results showed Native American ancestry with more variants than the other three ancestries in both cases and controls, which could be related to the great number of individuals from this ancestry in each group. Native American haplogroups were the most frequent in both case (36.5%) and control (44.6%). They were followed by European (27.3%), African (18.2%) and Asian (18.2%) in cases and by European and African (25.6% each) and Asian (4.2%) in controls. This ancestry profile based on mtDNA is consistent to the expected for the studied geographic region, considering the admixture process that formed the Brazilian population^26,48,49^. In addition, it is informative that there was a statistical significance in case group (P 0.047), probably due to the high frequency of Native American haplogroups in this group. This is especially interesting considering that GC is one of the most incident types of cancer in the studied Brazilian region, as previously mentioned.

Moreover, in both case and control groups, the most frequent haplogroup was C (Native American ancestry), but it is important to highlight that this haplogroup accounted for more than 2-fold in case group than in control group (36.4% and 17%, respectively). This suggests that haplogroup C might be related to gastric cancer development, which is reinforced by a study performed with Tibetan patients of gastric cancer, in which haplogroup C was the third most common (accounting for 17%)^50^.

Considering that the parent haplogroup of C is M^51^, it is also interesting that this haplogroup is present in our case group, but not in our control group. On the other hand, our study reported sibling haplogroup N in control group, but not in case group. Similarly, it has been suggested that haplogroup N is associated to good outcome of gastric cancer clinical evolution when compared to haplogroup M^18^. In addition, haplogroup H (European ancestry) was the second most common haplogroup in case group (18.2%). Although this haplogroup has not been previously associated to gastric cancer, it has been indicated that it might influence other types of cancer^52^.

## Conclusions

In this study, we were able to successfully sequence the whole mitochondrial genome of FFPE samples and associate it to gastric cancer development. To the best of our knowledge, this was the first study to achieve that. We found more mitochondrial variants in tumor group than in controls, suggesting that such variants and mitochondrial heteroplasmy might influence gastric cancer development. In addition, haplogroup C might also be important to the development of this type of cancer. Although limited by sample number, our findings contribute to a greater understanding of mitochondrial influence in gastric cancer. Further, functional studies are recommended to clarify the mechanism of this impact.

## Methods

### Sampling

In this study, we included formalin-fixed and paraffin-embedded (FFPE) tissue samples from patients going through gastric biopsy by endoscopy or surgical resection in the Unit of High Complexity in Oncology of the University Hospital João de Barros Barreto (UNACON/HUJBB-UFPA). Individuals with gastric cancer diagnosis had their samples collected before undergoing chemotherapy or radiotherapy. All samples were analyzed by a pathologist that confirmed the positive or negative diagnosis of cancer.

**Figure 7:**
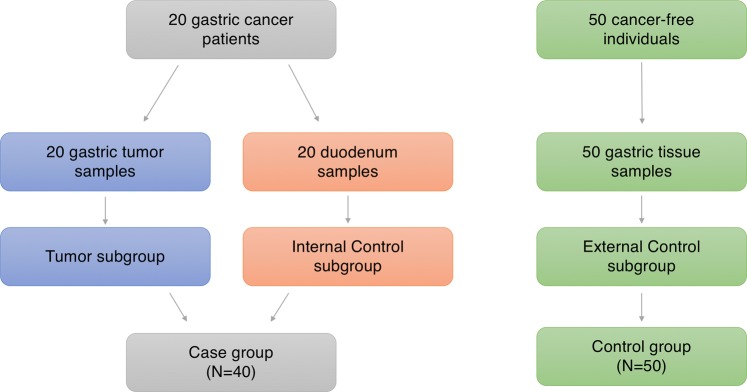
Schematic visualization of the sampling design.

We investigated 70 individuals (90 samples), divided in two main groups (Fig. 7). The first group (“Case”) consists of samples obtained from 20 individuals with gastric cancer diagnosis before treatment: gastric tumor tissue samples (“Tumor” subgroup) and non-tumor tissue of the duodenum (“Internal Control” subgroup) were collected from each patient, totalizing 40 samples in the Case group. Duodenal portion was extracted during gastrectomy and confirmed to be cancer-free by a pathologist. These samples were chosen as internal control respecting the cancerization field effect. The second group (“External Control”) is composed by gastric tissue samples from 50 individuals with no history of cancer and no infection by *Helicobacter pylori*.

### DNA extraction and quantification

The DNA extraction from FFPE samples was performed with DNA IQ™ System (Promega Corporation, Fitchburg, WI, USA), following manufacturer’s instructions. Quantification of DNA was performed with NanoDrop 1000 and Qubit™ dsDNA HS Assay Kit (Thermo Fisher Scientific, Wilmington, DE, USA).

### Mitochondrial genome amplification

From the extracted DNA, mtGenome was amplified by PCR with specific sets of primers (Supplementary Table S2). These sets were composed of 33 pairs of designed primers with discontinuous genomic location in order to provide overlaps in order to cover the whole fragmented genome.

### Mitochondrial genome sequencing

The mtGenome was sequenced in the MiSeq System (Illumina Inc., Chicago, IL, USA) with MiSeq Reagent Kit v3 (300-cycles) (Illumina). Preparation of libraries was done with Nextera XT DNA Library Preparation Kit (96 samples) (Illumina), according to manufacturer’s instructions. DNA quality during library preparation was assessed with High Sensitivity D1000 ScreenTape at Agilent 2200 TapeStation System (Agilent Technologies, Santa Clara, CA, USA).

### Data analysis

Paired-end sequencing reads (.fastq files) were aligned with the human reference mtDNA sequence – revised Cambridge reference sequence, rCRS – using Burrows-Wheeler Alignment tool (BWA)^53^. SAMtools^54^ were used for mapping and sorting sequences, while Picard^55^ was used to mark duplicated reads. After preprocessing sequences with the aforementioned steps, paired-end.bam files were submitted to mtDNA-Server analysis^56^ for heteroplasmy detection and haplogroup inferences. Phred quality scores (Q scores) of 20, 30 and 20 were considered for mapping quality of reads, alignment quality and base quality, respectively. For heteroplasmy detection at mtDNA-Server, we considered at least 10x of coverage on forward and reverse strands. Using its own pipeline, mtDNA-Server performed automatic mtDNA haplogroup classification using PhyloTree 17^51^, implemented with HaploGrep^57^.

Statistical analyses were performed using R language^58^ and the following tests were employed for different analyses: Mann-Whitney test, chi-squared test, one-way ANOVA with Tukey’s HSD test and Poisson regression. Statistical power was also assessed using R. R packages ggplot^59^, UpSet^60^ and RCircos^61^ were used for graphic representations. In all analyses, p-value was considered statistically significant when ≤0.05.

### Ethics approval and consent to participate

All participants signed an informed consent, with approval by the Committee for Research Ethics of Hospital João de Barros Barreto under Protocol No. CAAE: 64399617.7.0000.5634. All methods were conducted in accordance with the relevant guidelines and regulations.

## Supplementary information

Supplementary Table S1

Supplementary Table S2

Supplementary Table S3

## Data Availability

The datasets used and/or analyzed during the current study are available from the corresponding author on reasonable request.

## References

[1] Shaughnessy DT (2014). Mitochondria, Energetics, Epigenetics, and Cellular Responses to Stress. Environmental Health Perspectives.

[2] Rigoli L, Caruso RA (2014). Mitochondrial DNA alterations in the progression of gastric carcinomas: Unexplored issues and future research needs. World Journal of Gastroenterology.

[3] Taylor RW, Turnbull DM (2005). Mitochondrial DNA mutations in human disease. Nature Reviews Genetics.

[4] Lee J-H, Kim D-K (2014). Microsatellite Instability of Nuclear and Mitochondrial DNAs in Gastric Carcinogenesis. Asian Pacific Journal of Cancer Prevention.

[5] Neiman M, Taylor DR (2009). The causes of mutation accumulation in mitochondrial genomes. Proceedings of the Royal Society B: Biological Sciences.

[6] Lee H (2015). Is Mitochondrial DNA Copy Number Associated with Clinical Characteristics and Prognosis in Gastric Cancer?. Asian Pacific Journal of Cancer Prevention.

[7] Weigl S, Paradiso A, Tommasi S (2013). Mitochondria and Familial Predisposition to Breast Cancer. Current Genomics.

[8] Mori MP, Souza‐Pinto NCde (2018). Role of mitochondrial dysfunction in the pathophysiology of DNA repair disorders. Cell Biology International.

[9] Hanahan D, Weinberg RA (2011). Hallmarks of cancer: the next generation. Cell.

[10] Bianchi NO, Bianchi MS, Richard SM (2001). Mitochondrial genome instability in human cancers. Mutation Research/Reviews in Mutation Research.

[11] de Araujo LF (2015). Mitochondrial genome instability in colorectal adenoma and adenocarcinoma. Tumor Biology.

[12] Lin C-S, Wang L-S (2013). Mitochondrial DNA instability in human cancers. Formosan Journal of Surgery.

[13] Lee H-C (2005). Mitochondrial genome instability and mtDNA depletion in human cancers. Ann. N. Y. Acad. Sci..

[14] Machado AMD (2009). Helicobacter pylori Infection Induces Genetic Instability of Nuclear and Mitochondrial DNA in Gastric Cells. Clinical Cancer Research.

[15] He Y (2010). Heteroplasmic mitochondrial DNA mutations in normal and tumour cells. Nature.

[16] GLOBOCAN. Cancer today Available at, http://gco.iarc.fr/today/home (Accessed: 6th March 2019) (2018).

[17] INCA. INCA - Instituto Nacional de Câncer - Estimativa 2018. *INCA - Instituto Nacional de Câncer* Available at, http://www1.inca.gov.br/estimativa/2018/ (Accessed: 9th February 2019) (2018).

[18] Wang C, Wang Y, Wang H, Zhang R, Guo Z (2014). Mitochondrial DNA haplogroup N is associated good outcome of gastric cancer. Tumor Biology.

[19] Jorgenson E, Choquet H, Yin J, Asgari MM (2018). Common Mitochondrial Haplogroups and Cutaneous Squamous Cell Carcinoma Risk. Cancer Epidemiol Biomarkers Prev.

[20] Kim HR (2018). Spectrum of mitochondrial genome instability and implication of mitochondrial haplogroups in Korean patients with acute myeloid leukemia. Blood research.

[21] Luchini C (2018). Female-specific association among I, J and K mitochondrial genetic haplogroups and cancer: A longitudinal cohort study. Cancer Genetics.

[22] Pinheiro D (2014). Perspectives on new biomarkers in gastric cancer: diagnostic and prognostic applications. World J. Gastroenterol..

[23] Rodrigues-Antunes S, Borges BN (2019). Alterations in mtDNA, gastric carcinogenesis and early diagnosis. Mitochondrial DNA Part A.

[24] Swerdlow, R. H. *et al*. Chapter Nine - Mitochondria, Cybrids, Aging, and Alzheimer’s Disease. in *Progress in Molecular Biology and Translational Science* (ed. Reddy, P. H.) **146**, 259–302 (Academic Press, 2017). 10.1016/bs.pmbts.2016.12.017 PMC5864124 28253988

[25] Ju, Y. S. *et al*. Origins and functional consequences of somatic mitochondrial DNA mutations in human cancer. *Elife* **3** (2014). 10.7554/eLife.02935 PMC4371858 25271376

[26] Schaan AP (2017). mtDNA structure: the women who formed the Brazilian Northeast. BMC Evolutionary Biology.

[27] Betge J (2015). Amplicon Sequencing of Colorectal Cancer: Variant Calling in Frozen and Formalin-Fixed Samples. Plos One.

[28] Hedegaard J (2014). Next-Generation Sequencing of RNA and DNA Isolated from Paired Fresh-Frozen and Formalin-Fixed Paraffin-Embedded Samples of Human Cancer and Normal Tissue. PLoS ONE.

[29] Astolfi A (2015). Whole exome sequencing (WES) on formalin-fixed, paraffin-embedded (FFPE) tumor tissue in gastrointestinal stromal tumors (GIST). BMC Genomics.

[30] Araujo LF (2018). Mitochondrial genome analysis in penile carcinoma. Mol. Biol. Rep..

[31] Järviaho T (2018). Novel non-neutral mitochondrial DNA mutations found in childhood acute lymphoblastic leukemia. Clin. Genet..

[32] Grzybowska-Szatkowska L, Slaska B, Rzymowska J, Brzozowska A, Floriańczyk B (2014). Novel mitochondrial mutations in the ATP6 and ATP8 genes in patients with breast cancer. Mol Med Rep.

[33] Chintha R, Kaipa PR, Sekhar N, Hasan Q (2013). Mitochondria and tumors: a new perspective. Indian J Cancer.

[34] Tan D-J (2006). Significance of somatic mutations and content alteration of mitochondrial DNA in esophageal cancer. BMC Cancer.

[35] Zhou S (2007). Frequency and phenotypic implications of mitochondrial DNA mutations in human squamous cell cancers of the head and neck. Proc. Natl. Acad. Sci. USA.

[36] Urra, F. A., Muñoz, F., Lovy, A. & Cárdenas, C. The Mitochondrial Complex(I)ty of Cancer. *Front Oncol* **7** (2017). 10.3389/fonc.2017.00118 PMC5462917 28642839

[37] Nelson, D. L. & Cox, M. M. *Lehninger Principles of Biochemistry*. (Freeman, W. H. & Company, 2017).

[38] Liu Z-W (2017). High incidence of coding gene mutations in mitochondrial DNA in esophageal cancer. Mol Med Rep.

[39] Yin P-H (2010). Somatic mutations of mitochondrial genome in hepatocellular carcinoma. Mitochondrion.

[40] Mohamed Yusoff AA (2017). Detection of somatic mutations in the mitochondrial DNA control region D-loop in brain tumors: The first report in Malaysian patients. Oncol Lett.

[41] Yuan R-T, Sun Y, Bu L-X, Jia M-Y (2015). Gene mutations in the D-loop region of mitochondrial DNA in oral squamous cell carcinoma. Molecular Medicine Reports.

[42] Guo Z (2016). Identification of sequence polymorphisms in the D-Loop region of mitochondrial DNA as a risk factor for colon cancer. Mitochondrial DNA A DNA Mapp Seq Anal.

[43] Zhao Y-B, Yang H-Y, Zhang X-W, Chen G-Y (2005). Mutation in D-loop region of mitochondrial DNA in gastric cancer and its significance. World J. Gastroenterol..

[44] Wang Y (2017). Cancer risk associated single nucleotide polymorphisms of mitochondrial D-loop and 8-hydroxy-2’-deoxyguanosine levels in gastric cancer. Biotechnology & Biotechnological Equipment.

[45] Li W (2017). Heteroplasmy and Copy Number Variations of Mitochondria in 88 Hepatocellular Carcinoma Individuals. J Cancer.

[46] Wei L (2011). Association of mtDNA D-Loop Polymorphisms with Risk of Gastric Cancer in Chinese Population. Pathology & Oncology Research.

[47] Han C-B (2005). Mutations of mitochondrial 12S rRNA in gastric carcinoma and their significance. World J. Gastroenterol..

[48] Alves-Silva J (2000). The Ancestry of Brazilian mtDNA Lineages. The American Journal of Human Genetics.

[49] Marinho AN (2006). Paleogenetic and taphonomic analysis of human bones from Moa, Beirada, and Zé Espinho Sambaquis, Rio de Janeiro, Brazil. Memórias do Instituto Oswaldo Cruz.

[50] Jiang J (2015). Analysis of mitochondrial DNA in Tibetan gastric cancer patients at high altitude. Molecular and Clinical Oncology.

[51] van Oven M, Kayser M (2009). Updated comprehensive phylogenetic tree of global human mitochondrial DNA variation. Hum. Mutat..

[52] Bonilla C (2015). Breast cancer risk and genetic ancestry: a case-control study in Uruguay. BMC Womens Health.

[53] Li H, Durbin R (2009). Fast and accurate short read alignment with Burrows-Wheeler transform. Bioinformatics.

[54] Li H (2009). The Sequence Alignment/Map format and SAMtools. Bioinformatics.

[55] Broad Institute. Picard Tools - By Broad Institute. Available at, https://broadinstitute.github.io/picard/ (Accessed: 7th March 2019).

[56] Weissensteiner H (2016). mtDNA-Server: next-generation sequencing data analysis of human mitochondrial DNA in the cloud. Nucleic Acids Res..

[57] van Oven M (2015). PhyloTree Build 17: Growing the human mitochondrial DNA tree. Forensic Science International: Genetics Supplement Series.

[58] R Core Team. R: A language and environment for statistical computing (2014).

[59] Wickam, H. *ggplot2 - Elegant Graphics for Data Analysis*. (Springer-Verlag, 2016).

[60] Lex A, Gehlenborg N, Strobelt H, Vuillemot R, Pfister H (2014). UpSet: Visualization of Intersecting Sets. IEEE Transactions on Visualization and Computer Graphics.

[61] Zhang H, Meltzer P, Davis S (2013). RCircos: an R package for Circos 2D track plots. BMC Bioinformatics.

